# Prescription Monitoring Program Review Among Patients with Cancer Receiving Opioids at a Safety-Net Palliative Medicine Clinic

**DOI:** 10.3390/cancers18050762

**Published:** 2026-02-27

**Authors:** Soraira Pacheco, Linh M. T. Nguyen, Joseph A. Arthur, Christopher M. Manuel, Wei Qiao, David Hui

**Affiliations:** 1 Joan and Stanford Alexander Division of Geriatric and Palliative Medicine, The University of Texas Health Science Center at Houston, Houston, TX 77030, USA; 2 Department of Palliative Care, Rehabilitation and Integrative Medicine, MD Anderson Cancer Center, Houston, TX 77030, USA; 3 Department of Biostatistics, MD Anderson Cancer Center, Houston, TX 77030, USA; 4 Center for Goal Concordant Care Research, The University of Texas MD Anderson Cancer Center, Houston, TX 77030, USA; 5 Department of General Oncology, The University of Texas MD Anderson Cancer Center, Houston, TX 77030, USA

**Keywords:** analgesics, opioid, cancer pain, neoplasm, opioid-related disorders, illicit drugs, pain management, palliative care, monitoring

## Abstract

Introduction: Prescription monitoring programs (PMPs) are commonly used to monitor non-medical opioid use (NMOU); however, the effectiveness of PMPs for identifying cancer patients with risk factors is not well known. Methods: This study assessed the frequency and predictors of concerning PMP findings among cancer patients in a palliative care clinic and examined the ability of PMPs, clinical review, and urine drug testing to identify NMOU behaviors. This was a retrospective analysis of consecutive cancer patients seen by palliative care at a safety-net hospital over four years. Demographic, clinical, and psychosocial risk factors for NMOU were extracted from the medical record. Concerning PMP findings were based on prescriber documentation. Logistic regression models identified predictors of documented PMP concerns. Results: Among 906 patients, 844 (93%) had PMP reviews at either consultation or a follow-up visit. Of these, 31/844 (4%) demonstrated documented PMP concern. Predictors of documented PMP irregularities included a history of illicit drug use (OR 6.30, 95% CI: 2.35–17.06), opioid use for non-malignant pain (OR 19.49, 95% CI: 6.24–60.90), and a family history of illicit drug use (OR 5.42, 95% CI: 0.96–25.04). Discussion: A total of 166 patients (20%) were identified as having NMOU behaviors based on clinical review; in contrast, PMP review identified only 31 (4%) patients with NMOU behaviors, and two (6%) were missed by clinical review. Documented PMP concern was low in cancer patients. Clinical review identified most patients with NMOU behaviors, with limited contribution from PMP review. Our findings suggest that PMP should not be used in isolation when assessing opioid-related risk in this population.

## Simple Summary

Patients with cancer frequently require opioid therapy for pain management and are at risk for non-medical opioid use. Prescription monitoring programs are often used to track patients’ prescribed opioids and to promote safe opioid use; however, their utility in patients with cancer is uncertain. In this study, we aimed to determine the prevalence of NMOU behaviors and examine the effectiveness of prescription monitoring program reviews compared to chart review and/or urine drug testing concerning opioid-related behaviors in cancer patients at a safety-net palliative medicine clinic. We found that abnormal findings on prescription monitoring program review were uncommon and identified far fewer patients with concerning behaviors than clinical review and urine drug screen. These results suggest that prescription monitoring programs alone may miss important signs of non-medical opioid use in patients with cancer. Our findings highlight the importance of comprehensive clinical evaluation when monitoring opioid safety in cancer care.

## 1. Introduction

Approximately 65% of cancer patients report experiencing cancer pain, which significantly impacts their function and quality of life [1]. Opioids are the gold standard for treatment of cancer-related pain; however, long-term opioid use carries a risk of non-medical opioid use (NMOU) [2,3,4,5,6,7]. NMOU is defined as using opioids in greater amounts, more often, or longer than prescribed, or for a reason other than a doctor’s instructions. NMOU, particularly opioid-use disorder, is associated with significant distress and overdose deaths [7]. Due to the opioid epidemic in the USA, several strategies and policies, including urine drug tests (UDT), patient-prescriber agreements (PPA), and prescription monitoring program (PMP) review, have been developed to mitigate NMOU behaviors [7,8,9,10,11,12,13].

PMPs are state-operated electronic databases that collect and monitor information on dispensed Schedule II, III, IV, and V controlled substances. Pharmacies in all 50 states and the District of Columbia are required to report all dispensed controlled substances to their state’s PMP, typically no later than the next business day after filling the prescription [14,15]. However, national standards for data collection are lacking; the specific data collected (such as the schedules of controlled substances included, prescriber information, dispensing pharmacy details, patient diagnoses, and payment methods) varies by state. PMP review is mandatory for prescribing controlled substances in at least 40 states as of today [14,15].

Currently, there is insufficient evidence to support that mandatory PMP review leads to a reduction in NMOU behaviors. Studies in non-cancer populations have yielded mixed results regarding PMPs’ effectiveness in reducing inappropriate opioid-prescribing or adverse outcomes such as opioid-use disorder and overdose [16,17,18,19,20,21]. In the oncology setting, only a few small studies have evaluated integrating PMP into practice in regard to prescribing practices and pain control [22,23,24,25]. To our knowledge, the studies have not specifically examined how effective PMP was at identifying NMOU behaviors in cancer patients such as early refills or multiple prescribers. Some studies suggest that the implementation of PMPs has inadvertently led to reduced opioid prescriptions for cancer patients, but did not measure or assess NMOU behaviors [23,25,26,27].

A clearer understanding of how PMP review can identify NMOU behaviors in cancer patients on long-term opioids could guide the integration of these measures into clinical practice. This study examined PMP reviews and documented irregularities detected in PMP reviews within a palliative cancer care clinic. Specifically, we assessed the frequency and predictors of documented PMP concern and evaluated its practical utility in identifying patients with NMOU behaviors compared to clinical assessments.

## 2. Materials and Methods

### 2.1. Design

This is a pre-planned secondary analysis of a retrospective study examining the utility of strategies to support safe opioid use [7,12,13]. The current study focuses on the pattern of PMP use and its ability to detect NMOU. The eligibility criteria have been published previously [7,12,13]. Briefly, we included consecutive patients with cancer who were seen at a palliative care clinic for consultation at Harris Health Lyndon B. Johnson (LBJ) Hospital in Houston, TX, between 1 September 2015, and 31 December 2019. LBJ is a safety-net hospital that serves predominantly low-income and uninsured patients. Patients were included if they were 18 years or older, had a diagnosis of cancer, and received opioid prescriptions at any time during the study period. We excluded patients with non-cancer diagnoses and those who only saw us once in the clinic. This study protocol has been approved by the Institutional Review Board at McGovern Medical School and Harris Health, with a waiver of informed consent.

### 2.2. Data Collection

We collected patients’ demographic information, such as age, sex, race, ethnicity, marital status, cancer diagnosis, and stage, as well as risk factors for NMOU, such as a history of substance use disorder, marijuana use, tobacco use, and alcoholism [6,28]. We also assessed psychiatric comorbidities and a family history of substance use disorder. Additionally, we collected data on symptom burden using the Edmonton Symptom Assessment Scale (ESAS). ESAS measures the average symptom intensity in the past 24 h using a 0–10-point numeric rating scale, where 0 = no symptom and 10 = worst possible (such as pain, fatigue, nausea, depression, anxiety, drowsiness, shortness of breath, appetite, sleep, feeling of well-being, constipation, family distress, financial distress, spiritual pain) [19,29].

We collected information regarding PMP documentation (whether completed or not) from our electronic health records. If the PMP was completed, we collected the date, whether there was any PMP concern was listed in our notes as “aberrancy”, and noted (yes/no). We also collected the reasons for PMP concern, including having multiple prescribers, early refills, or not filling prescriptions. As part of our clinic protocol, physicians were asked to review and document PMP review findings in our electronic health record. During the PMP review, providers could view whether opioids were filled and dispensed, along with the date, quantity of medication, prescriber, and the hospital or clinic associated with the prescriber. Upon review of the PMP website, each prescribing physician documented whether there was a PMP concern based on their clinical judgment.

We also collected data on immunoassay UDT from the electronic health record. We documented whether UDT was performed (yes/no) and the presence or absence of amphetamine, barbiturate, benzodiazepine, cannabinoid, cocaine, opiate, and PCP. Aberrancy (yes/no) was defined as the presence of unprescribed opioids, the absence of prescribed opioids, positive screens for amphetamine, barbiturates, unprescribed benzodiazepines, cocaine, or PCP. Details of UDT, coding for aberrancy, and limitations have been reported previously [13]. Similar to previous studies, UDT was not considered to be aberrant if it was only positive for marijuana but otherwise normal [7,13]. We excluded marijuana since state laws regarding marijuana vary significantly, with most states legalizing marijuana for medical and/or recreational use despite federal prohibition [30].

Behaviors indicating NMOU were collected by reviewing patient charts from consultations and follow-up visits at our palliative care clinic. We collected information on the following NMOU behaviors (present/absent) and determined whether they were aberrant (yes/no): excessive self-increase in dose, running out of opioids early, unable to provide UDT, any aberrant UDT findings (except marijuana use), use of illicit drugs (except marijuana use), lost or stolen opioids, resisting changes to opioid regimen, obtaining opioids from unauthorized sources, not following directions regarding pain medications, not following directions regarding referrals, using pain medications for reasons other than pain, and other reasons.

### 2.3. Statistical Analysis

We summarized patient characteristics using descriptive measures, including counts, percentages, means, standard deviations, medians, and interquartile ranges. Fisher’s exact test was used to assess the association between categorical variables and PMP documentation status, while the Wilcoxon test was used to evaluate the association between numerical variables and PMP documentation status. For model selection purposes, we first conducted univariable regression modeling, with the aberrancy based on the PMP as the outcome (yes/no). Variables that had a *p*-value of <0.20 were then included in a model selection process, where the model with the lowest AIC was selected. We forced the pain at consult and Hispanic ethnicity variables into the models regardless of the insignificance in the univariable setting due to research interest. R was used for regression modeling purposes. Finally, we presented the results of the model multivariable logistic regression with the lowest AIC, estimated using the Firth penalty. For each variable in the regression models, we reported the odds ratio (OR), *p*-value, 95% confidence intervals, and also included the Nagelkerke R^2^ and overall model *p*-values. The following variables considered included: Hispanic (any race), single marital status, illicit drug use other than marijuana, marijuana use, tobacco use, family history of illicit drug use, personal history of criminal activity other than marijuana, contact with persons involved in criminal activity other than marijuana, pain presentation inconsistent with history and physical, and use of opioids for non-malignant pain. We reported *p*-values and the 95% confidence interval bands to four decimal places to avoid potential misinterpretations. The frequency and percentage of reasons for documented PMP concern were also evaluated. The McNemar test was used to examine differences in the rates of identifying aberrancy among NMOU behaviors, UDT, and documented PMP concern. We conducted a sensitivity analysis using a dataset where PMP concern is based on PMP status reported in any follow-up visit within the first six months of the baseline visit.

Statistical analyses were conducted using STATA SE version 17.0 (College Station, TX, USA) and R version 4.4.1. A *p*-value of less than 0.05 was considered statistically significant.

## 3. Results

A total of 906 consecutive patients met the eligibility criteria and were included in the study. The median age was 56 (range 18–93). Of those patients, 473 (52%) were female, 420 (46%) were of Hispanic ethnicity, 605 (67%) were single, and 609 (67%) had metastatic cancer. Demographics at the time of consultation can be seen in Table 1. Of the patients seen in the palliative care clinic, 809 (89%) had a documented PMP review at the initial consultation; 844 patients had a documented PMP review at either the consultation or a follow-up visit. White non-Hispanic patients were less likely to undergo PMP review, while Black non-Hispanic patients were more likely to undergo PMP review (*p* = 0.025) (Table 1). There was no significant difference in median age, marital status, or cancer stage between patients with and without PMP review.

Of the measured NMOU risk factors, a history of tobacco use was significantly associated with PMP review (*p* = 0.02) but not the other risk factors. The risk factors for NMOU at baseline are shown in Table 2.

In the univariable analysis, Hispanic patients had lower odds of having documented PMP concern (OR 0.32, 95% CI: 0.12–0.85, *p* = 0.02). Patients with known risk factors for NMOU had higher odds (Table 3). In our multivariable analysis, several NMOU risk factors were associated with documented PMP concern. A history of illicit drug use was strongly associated (OR 6.30, 95% CI: 2.35–17.06, *p* < 0.001), as was opioid use for non-malignant pain (OR 19.49, 95% CI: 6.24–60.90, *p* < 0.001). A family history of illicit drug use showed a suggestive association with documented PMP concern (OR 5.42, 95% CI: 0.96–25.04, *p* = 0.04). In addition, higher MEDD was modestly associated with documented PMP concern (OR 1.06 per 10 mg increase, 95% CI: 1.01–1.10, *p* = 0.01). To assess if time-window mismatch affected our outcomes, we assessed patients that had follow up visits with any frequency (including >6 months) vs. patients with follow up visits only within six months. Then, we performed sensitivity analysis to compare and found prevalence was similar (2.4% within 6 months compared to 2.8% for all follow up visits). The results of the multivariable modeling can be found in Table 3 and Table 4 (visits within the first six months), and the results using different time windows are relatively consistent.

Among the 844 patients who had a documented PMP review at either consultation or follow-up, 31 (4%) documented PMP concern at any point. Of these, 25 patients were identified as having PMP concerns at the initial consultation. The most common reasons for documented PMP concern were having multiple prescribers (*n* = 39, 83%), obtaining early refills (*n* = 7, 15%), and not filling prescriptions (*n* = 1, 2%). We examined the NMOU behaviors detected by clinical review plus UDT, UDT alone, and PMP review (Table 5). Among the 844 patients with documentation of all three strategies, 166 patients (20%) were identified as aberrant through clinical review plus UDT, and 99 patients (12%) were identified as aberrant through UDT alone. In contrast, PMP review yielded only 31 patients with documented PMP concern (4%). PMP review identified only two new patients with concerning behaviors that were not already captured by clinical documentation of NMOU behaviors and UDT review.

## 4. Discussion

Among the substantial majority of patients (89%) who underwent PMP review at consult, only a small proportion (4%) were identified as having concerning behaviors based solely on PMP review. We found that Black patients were significantly more likely to have had a documented PMP review. Patients with NMOU risk factors and higher MEDD were significantly more likely to have documented PMP concerns. Importantly, PMP alone missed a large number of patients who exhibited NMOU behaviors and only identified a few patients not already captured by the clinical review and UDT. These findings may help refine the role of PMP within clinical practice.

In our study, PMP data review identified concerning findings in only 4% of patients. This small proportion is not unexpected given that PMP only provides information on prescription patterns. Consistent with our study, previous studies conducted primarily in emergency department populations have reported PMP sensitivities for detecting at risk behavior of 7% to 36% [31,32]. The limited sensitivity of PMPs likely reflects their dependence on prescription-based indicators—such as elevated morphine equivalent daily dose, multiple prescribers or dispensing pharmacies, and concurrent prescriptions for other controlled substances (e.g., benzodiazepines). PMP data can capture prescribing patterns but it does not capture intent (e.g., multiple prescribers due to fragmented care or undertreated pain) nor does it encompass broader behavioral, psychosocial, or familial risk factors that contribute to NMOU. Taken together, our findings underscore the important limitations of PMP and the need to review clinical history.

The Centers for Disease Control and Prevention (CDC), American Society of Clinical Oncology (ASCO), and National Comprehensive Cancer Network (NCCN) support the regular review of PMPs, especially since it is mandated in many states [6,9,33]. The high rate of PMP review observed in our clinical practice is broadly consistent with established guidelines [6,9,33]. Nevertheless, our analysis identified a significant racial and ethnic disparity in PMP review frequency in our clinic. Black non-Hispanic patients underwent review more often, whereas White non-Hispanic patients were reviewed less frequently by our providers. Observed differences may be attributable to variation in clinician practice and documentation patterns and may also reflect unmeasured sources of implicit bias, highlighting the importance of standardized PMP review workflow to promote equitable care. Several studies have shown that PMP review may result in a more pronounced reduction in opioid dispensing among Black patients compared to White patients, despite Black patients exhibiting lower baseline opioid dispensing rates [34,35,36,37,38]. Further studies are needed to examine strategies to reduce racial disparities.

Patients with known risk factors for NMOU had significantly higher odds of having documented PMP concern. This is not unexpected and is consistent with prior literature. Although limited data exist regarding predictors of PMP review irregularities specifically, several factors have been associated with doctor shopping or multiple-provider episodes in other populations. These include younger age (<50 years), psychiatric comorbidities such as anxiety, bipolar disorder, or post-traumatic stress disorder, a history of alcohol misuse, prior or active drug use, and other substance use disorders [31,32,39,40,41,42].

To our knowledge, this is the first study to evaluate PMP data review in a cancer population and to directly compare it with UDT results and documented NMOU risk factors. Clinical review for NMOU risks and UDT are inherently imperfect, may be influenced by clinician beliefs and documentation practices, and may result in false positive or negative findings; however, they have been extensively studied and represent widely accepted measures for assessing opioid-related risk [5,6,7,8,9,10,11,12,13]. Among 813 patients with documentation of all three strategies, 166 patients (20%) were identified as aberrant based on clinical documentation and UDT but these were not picked up by PMP. In contrast, PMP review alone only identified two patients not otherwise found to have aberrant behaviors with NMOU behavior review and UDT. These findings suggest the importance of clinical history and UDT in identifying patients with NMOU and that reliance on PMP data alone will substantially underestimate the prevalence of opioid misuse. A multimodal approach incorporating NMOU risk factors, clinical behavioral risk review, and objective testing may provide a comprehensive evaluation of NMOU behaviors.

Proponents of PMP review suggest that this practice may occasionally identify opioid misuse or diversion not evident through routine clinical assessment, thereby offering incremental value for risk mitigation [16,18,20,31,43]. Universal review of PMP data in cancer pain management is a simple, noninvasive intervention that can be systematically implemented with minimal disruption once integrated into the electronic health record. PMP checks can efficiently reveal patients obtaining prescriptions from multiple providers. However, this process introduces additional clinician time burden, and inconsistent use may contribute to prescription bias [14,16,24,26,34,36]. As demonstrated in this study, PMP alerts may fail to detect some high-risk patients while incorrectly flagging others as having irregularities, raising questions about their clinical utility [19,21,31]. Currently, evidence that universal PMP review prevents overdose or meaningfully influences opioid prescribing in cancer pain remains limited, and existing guidelines recommend its use only in those with high risk for NMOU [6,22,23,25,26,27,33].

This study offers several strengths, including its relatively large and ethnically diverse sample derived from consecutive patient encounters within a cancer palliative clinic at a safety-net hospital. Nonetheless, several limitations should be noted. Data was collected solely from one hospital system between September 2015 and December 2019, limiting its generalizability. Since data collection ended six years ago, substantial changes have occurred in the healthcare system, including integration of the PMP site into the electronic health record with mandatory PMP review for non-cancer patients in our state, and the COVID19 pandemic which increased the use of telehealth visits and remote opioid prescribing. Review of more recent data may be warranted. Our findings may not be generalizable beyond our setting, particularly in other countries where prescribing practices, regulatory frameworks, and the availability and scope of PMPs vary substantially. Despite a relatively high prevalence of NMOU in our clinic population, PMP review demonstrated limited sensitivity. In settings with a lower prevalence of NMOU, the rationale for mandating PMP review for cancer patients may be less compelling. Notably, our study underscores that a thorough clinical review can effectively identify NMOU behaviors, suggesting that good clinical practices may be more universally applicable than opioid data surveillance tools. Furthermore, the retrospective study design precluded our ability to directly observe and quantify the specific decision-making points influenced by PMP data review and we were also unable to control for confounding variables that might have impacted provider behavior.

## 5. Conclusions

In summary, this study adds to the literature on the utility of PMP and its ability to identify NMOU behaviors. Our study highlights that interpretation of PMP data should occur within the broader clinical context. Sole reliance on database findings without consideration of the patient’s pain history, disease status, and psychosocial factors can result in misinterpretation, inadequate pain control, or stigmatization. Therefore, PMP review should complement, rather than replace, comprehensive clinical judgment in evaluating opioid-related risk. Further research is needed to determine how to best integrate PMP with other risk mitigation measures to improve the detection of NMOU and promote equitable, patient-centered opioid stewardship without compromising access to appropriate pain management in cancer care.

## Figures and Tables

**Table 1:** Patient demographics and Edmonton Symptom Assessment System by Prescription Monitoring Program review status at consultation.

		PMP Review at Consultation		
Demographic Characteristics	Total *n* = 906 (%) *	No *n* = 97 (11%)	Yes *n* = 809 (89%)	*p*-Value
Age (in years), Median (range)	56 (18–93)	57 (30–93)	56 (18–89)	0.11
Sex, *n*				
Male	433 (48)	45 (46)	388 (48)	0.83
Female	473 (52)	52 (54)	421 (52)	
Race and Ethnicity				
White, Non-Hispanic	204 (23)	32 (33)	172 (21)	0.025
Black, Non-Hispanic	259 (29)	18 (19)	241 (30)	
Hispanic, any race	420 (46)	45 (46)	375 (46)	
Other race, Non-Hispanic	23 (3)	2 (2)	21 (3)	
Marital Status				
Married	301 (33)	39 (40)	262 (32)	0.14
Single **	605 (67)	58 (60)	547 (68)	
Cancer type				
Gastrointestinal	312 (35)	26 (27)	286 (35)	0.05
Respiratory	108 (12)	19 (20)	89 (11)	
Gynecological	105 (12)	12 (12)	93 (12)	
Genitourinary	94 (10)	15 (16)	79 (10)	
Breast	90 (10)	7 (7)	83 (10)	
Head and neck	84 (9)	10 (10)	74 (9)	
Hematological/Other	113 (12)	8 (8)	105 (13)	
Cancer stage: metastatic				
Not metastatic	297 (33)	33 (34)	264 (33)	0.87
Metastatic	609 (67)	64 (66)	545 (67)	
ESAS at consult only, median				
Pain	6	5	6	0.21
Tiredness	6	6	6	0.80
Nausea	0	0.5	0	0.69
Depression	0	0	1	0.71
Anxiety	1	1	1	0.56
Drowsiness	1	0	2	0.93
Appetite	4	4	4	0.56
Well-being	4	3	4	0.90
Shortness of breath	1	1	1	0.56
Family distress	0	0	0	0.39
Spiritual distress	0	0	0	0.68
Constipation	1	0.5	1	0.84
Sleep	5	5	5	0.43

* Unless otherwise specified. ** Single category includes unmarried, divorced, separated, and widowed. Abbreviations: Prescription Monitoring Program, PMP; ESAS, Edmonton Symptom Assessment System. *p* < 0.20 were included in the AIC multivariable.

**Table 2:** Clinical risk factors for non-medical opioid use at consultation.

		PMP Review at Consultation		
Characteristics	Total *n* = 906 (%) *	No *n* = 97 (11%)	Yes *n* = 809 (89%)	*p*-Value
NMOU Risk Factors				
History of illicit drug ***	160 (18)	19 (20)	141 (17)	0.57
History of marijuana use	197 (22)	19 (20)	178 (22)	0.70
History of tobacco use	456 (50)	59 (61)	397 (49)	0.02
History of excessive alcohol use	173 (19)	20 (21)	153 (19)	0.68
Psychiatric depression on meds	158 (18)	15 (16)	143 (18)	0.67
Family history of illicit drug use	17 (2)	1 (1)	16 (2)	>0.99
Personal history of criminal activity	84 (9)	12 (13)	72 (9)	0.26
Contact with persons involved in criminal activity	58 (6)	8 (8)	50 (6)	0.38
Inconsistent pain presentation	15 (2)	2 (2)	13 (2)	0.67
Use for non-malignant pain	36 (4)	4 (4)	32 (4)	0.79

* Unless otherwise specified. *** Excluding marijuana. Abbreviations: Prescription Monitoring Program, PMP; NMOU, non-medical opioid use. *p* < 0.20 were included in the AIC multivariable.

**Table 3:** Univariate and multivariable analysis of clinical risk factors for non-medical opioid use, all visits.

	Univariate Analysis				Multivariable Analysis	
	Unadjusted Odds Ratio (95% Confidence Interval)	*p*-Value	R^2^	Model *p*-Value	Adjusted Odds Ratio (95% Confidence Interval)	*p*-Value
Age	0.98 (0.95–1.00)	0.1314	0.0098	0.1355	0.97 (0.93–1.01)	0.1011
Sex: Male	Ref					
Female	0.74 (0.36–1.53)	0.4222	0.0028	0.4209		
Race-ethnicity: White, Non-Hispanic	Ref					
Hispanic any race	0.32 (0.12–0.85)	0.0239	0.0335	0.0532	0.64 (0.19–2.10)	0.4541
Non-White, Non-Hispanic	0.97 (0.42–2.29)	0.9357	-	-	1.04 (0.39–2.93)	0.9325
Black, Non-Hispanic	0.98 (0.42–2.34)	0.9585	-	-	-	-
Other race, Non-Hispanic	0.84 (0.04–4.71)	0.8694	-	-	-	-
Marital status: Married	Ref					
Single **	2.56 (1.05–7.62)	0.0573	0.0191	0.0369	-	-
Cancer type: Gastrointestinal	Ref					
Respiratory	1.55 (0.47–4.50)	0.4312	0.0258	0.4356	-	-
Gynecological	1.19 (0.32–3.65)	0.7714	-	-	-	-
Genitourinary	1.79 (0.54–5.20)	0.3000	-	-	-	-
Breast	0.67 (0.10–2.62)	0.6148	-	-	-	-
Head and neck	1.53 (0.41–4.72)	0.4829	-	-	-	-
Cancer stage: Non-metastatic	Ref					
Metastatic	0.76 (0.37–1.63)	0.4596	0.0023	0.4645	-	-
Risk factors of NMOU						
History of illicit drug use *	6.15 (2.97–12.97)	<0.001	0.0973	<0.0001	5.70 (1.88–17.79)	0.0021
History of marijuana use	4.00 (1.93–8.34)	<0.001	0.0583	0.0003	1.92 (0.73–5.20)	0.1867
History of tobacco use	2.52 (1.18–5.84)	0.0212	0.0255	0.0157	0.75 (0.28–2.01)	0.5603
History of alcohol use	2.07 (0.92–4.38)	0.0651	0.0136	0.0779	-	-
History of depression	1.33 (0.52–3.00)	0.5134	0.0018	0.5237	-	-
Family history of illicit drug use	6.59 (1.45–21.90)	0.0048	0.0243	0.0184	5.31 (1.03–23.00)	0.0463
Personal history of criminal activity	3.10 (1.2–7.10)	0.0115	0.0230	0.0219	-	-
Contact with persons involved in criminal activity	3.91 (1.40–9.44)	0.0044	0.0277	0.0118	-	-
Inconsistent pain presentation	11.90 (3.11–38.14)	<0.001	0.0473	0.0010	-	-
Use for non-malignant pain	10.96 (4.25–26.17)	<0.001	0.0864	<0.000	14.15 (3.94–50.24)	<0.001
MEDD in units of 10	1.04 (0.99–1.08)	0.0541	0.0131	0.0836	1.05 (1.00–1.10)	0.0313

* Excluding marijuana, ** Single category includes unmarried, divorced, separated, and widowed. Abbreviations: NMOU, non-medical opioid use; MEDD, morphine equivalent daily dose. The Nagelkerke R^2^ is 0.3447, and the model *p*-value is <0.001. *p* < 0.20.

**Table 4:** Univariate and multivariable analysis of clinical risk factors for non-medical opioid use at six months.

	Univariate Analysis				Multivariable Analysis	
	Unadjusted Odds Ratio (95% Confidence Interval)	*p*-Value	R^2^	Model *p*-Value	Adjusted Odds Ratio (95% Confidence Interval)	*p*-Value
Age	0.98 (0.95–1.00)	0.1314	0.0098	0.1355	0.95 (0.91–0.99)	0.0284
Sex: Male	Ref					
Female	0.74 (0.36–1.53)	0.4222	0.0028	0.4209	-	-
Race-ethnicity: White, Non-Hispanic	Ref					
Hispanic any race	0.32 (0.12–0.85)	0.0239	0.0335	0.0532	0.0334	0.0218
Non-White, Non-Hispanic	0.97 (0.42–2.29)	0.9357	-	-	1.48 (0.50–4.74)	0.4899
Black, Non-Hispanic	0.98 (0.42–2.34)	0.9585	-	-	-	-
Other race, Non-Hispanic	0.84 (0.04–4.71)	0.8694	-	-	-	-
Marital status: Married	Ref					
Single **	2.56 (1.05–7.62)	0.0573	0.0191	0.0369	-	-
Cancer type: Gastrointestinal	Ref					
Respiratory	1.55(0.47–4.50)	0.4312	0.0258	0.4356	-	-
Gynecological	1.19(0.32–3.65)	0.7714	-	-	-	-
Genitourinary	1.79(0.54–5.20)	0.3000	-	-	-	-
Breast	0.67(0.10–2.62)	0.6148	-	-	-	-
Head and neck	1.53 (0.41–4.72)	0.4829	-	-	-	-
Cancer stage: Non-metastatic	Ref					
Metastatic	0.76 (0.37–1.63)	0.4596	0.0023	0.4645	-	-
Risk factors of NMOU						
History of illicit drug use *	6.15 (2.97–12.97)	<0.001	0.0973	<0.0001	7.61 (2.18–28.74)	0.0014
History of marijuana use	4.00 (1.93–8.34)	<0.001	0.0583	0.0003	2.51(0.88–7.52)	0.0872
History of tobacco use	2.52 (1.18–5.84)	0.0212	0.0255	0.0157	0.94 (0.32–2.92)	0.9156
History of alcohol use	2.07 (0.92–4.38)	0.0651	0.0136	0.0779	0.41 (0.12–1.35)	0.1475
History of depression	1.33 (0.52–3.00)	0.5134	0.0018	0.5237	-	-
Family history of illicit drug use	6.59 (1.45–21.90)	0.0048	0.0243	0.0184	6.17(1.08–31.70)	0.0413
Personal history of criminal activity	3.10 (1.2–7.10)	0.0115	0.0230	0.0219	0.83(0.17–3.52)	0.8039
Contact with persons involved in criminal activity	3.91 (1.40–9.44)	0.0044	0.0277	0.0118	0.78 (0.14–3.99)	0.7687
Inconsistent pain presentation	11.90 (3.11–38.14)	<0.001	0.0473	0.0010	1.09 (0.16–7.00)	0.9215
Use for non-malignant pain	10.96 (4.25–26.17)	<0.001	0.0864	<0.0001	35.16 (8.36–161.03)	<0.001
MEDD in units of 10	1.04 (0.99–1.08)	0.0541	0.0131	0.0836	1.07 (1.02–1.11)	0.0107

* Excluding marijuana, ** Single category includes unmarried, divorced, separated, and widowed. Abbreviations: NMOU, non-medical opioid use; ESAS, Edmonton Symptom Assessment System. The Nagelkerke R^2^ is 0.3447, and the model *p*-value is <0.001.

**Table 5:** Comparison of non-medical opioid use behaviors as detected by Prescription Monitoring Program, clinical review plus urine drug test, and urine drug test alone.

	Documented PMP Concerns		
Total *n* = 844	No *n* = 813 (%)	Yes *n* = 31 (%)	*p*-Value
Aberrant NMOU behaviors * or aberrant UDT **			
Absent	647 (80)	2 (6)	<0.001
Present	166 (20)	29 (94)	
Aberrant UDT **			
Absent	714 (88)	13 (42)	<0.001
Present	99 (12)	18 (58)	

* Based on documented clinical chart review of aberrant behaviors by clinicians. ** Based on immunoassay UDT findings. Positivity for marijuana without other aberrant findings was not considered as aberrant in this study. Abbreviations: Prescription Monitoring Program, PMP; NMOU, non-medical opioid use; UDT, urine drug test.

## Data Availability

The data presented in the study are available on request from the corresponding author, S.P. The data are not publicly available due to their containing information that could compromise the privacy of research subjects.
